# Fast, parallel, and cache-friendly suffix array construction

**DOI:** 10.1186/s13015-024-00263-5

**Published:** 2024-04-28

**Authors:** Jamshed Khan, Tobias Rubel, Erin Molloy, Laxman Dhulipala, Rob Patro

**Affiliations:** Department of Computer Science, University of Maryland, College Park, MD 20742 USA

**Keywords:** Suffix array, Longest common prefix, Data structures, Indexing, Parallel algorithms

## Abstract

**Purpose:**

String indexes such as the suffix array (sa) and the closely related longest common prefix (lcp) array are fundamental objects in bioinformatics and have a wide variety of applications. Despite their importance in practice, few scalable parallel algorithms for constructing these are known, and the existing algorithms can be highly non-trivial to implement and parallelize.

**Methods:**

In this paper we present caps-sa, a simple and scalable parallel algorithm for constructing these string indexes inspired by samplesort and utilizing an LCP-informed mergesort. Due to its design, caps-sa has excellent memory-locality and thus incurs fewer cache misses and achieves strong performance on modern multicore systems with deep cache hierarchies.

**Results:**

We show that despite its simple design, caps-sa outperforms existing state-of-the-art parallel sa and lcp-array construction algorithms on modern hardware. Finally, motivated by applications in modern aligners where the query strings have bounded lengths, we introduce the notion of a bounded-context sa and show that caps-sa can easily be extended to exploit this structure to obtain further speedups. We make our code publicly available at https://github.com/jamshed/CaPS-SA.

## Introduction

Methods for aligning sequencing reads to reference genomes underlie some of the most well-developed and widely-used tools in bioinformatics [2]. Modern read-to-reference aligners typically employ an *index* over the reference text. A classic index for strings is the suffix array (sa) [40], which is an array of indices of the lexicographically sorted suffixes of a string. In alignment, the sa index is used by the popular STAR aligner [14] as well as in other tools [53, 55]. The sa has also been used in short-read error correction [24] and sequence clustering [23]. A related object frequently used in conjunction with the sa is the Longest Common Prefix (lcp) array, which contains the lengths of the longest shared prefixes between pairs of successive indices in the sa. For instance, the sa can be used in concert with the lcp-array (and other auxiliary tables derived from these) in a data structure called an enhanced suffix array [1] to mimic the functionality of a suffix tree [54], but often more efficiently and using less space. An account of the pervasiveness of the sa and the lcp-array in computational genomics is best left to a dedicated review (see e.g. [51]).

Because of the utility of the sa (and the lcp-array) in string indexing, significant work has been dedicated to developing practical algorithms for its construction. It is well-established that sa and lcp-array construction can be performed sequentially in time linear to the size of strings. However, as modern genomics pipelines produce ever more data—including more complete reference genomes and pangenomes—there has been a concerted effort to improve the practical efficiency and reduce the runtime of sa and lcp-array construction. A host of efficient serial algorithms have been developed [18, 30, 34, 35, 38, 42, 44]. Likewise, in an effort to take advantage of the increased parallelism of modern computer hardware, a number of parallel algorithms have also been proposed; e.g. parallel DivSufSort [37], parallel DC3 [36], and parallel divide-and-conquer based sa-construction [28]. External memory algorithms [26, 27, 29] have also been a focus of recent research because of the memory bottlenecks that arise when building the sa and the lcp-array on genomic datasets. Besides, algorithms for GPU-settings [39] and distributed-memory [19, 20] have been developed. We refer the interested reader to [5, 6, 48] for a comprehensive review. For our purposes, we note that these increasingly advanced methods introduce new algorithmic techniques to enable parallelism or improve the worst-case time complexity (so that it is sublinear). The trade-off, often, is that these more complex algorithms may potentially be more difficult to implement, optimize for modern hardware and cache layouts, and to maintain.

In this work, we address these issues by introducing caps-sa [33], a highly parallel method for constructing the sa and the lcp-array. A core principle behind caps-sa is simplicity. Our approach draws on several existing algorithms and techniques, and focuses on their efficient combination for the problem of highly parallel sa construction. The algorithm builds upon the *parallel samplesort* algorithm [21], and is easy to implement and optimize for modern hardware.

A potential downside of our approach is that it is *output-sensitive* and as a result its worst-case time complexity on adversarial inputs is quadratic. However, in practice we find that the shared-memory implementation of caps-sa outperforms state-of-the-art methods (specifically parallel-divsufsort  [37] and parallel-dc3  [3, 36]) in terms of runtime and scalability (although not in memory for Parallel-divsufsort). For example, caps-sa can build the sa and the lcp-array for the telomere-to-telomere human genome assembly (CHM13 v2) [45] in 106 s using 48 GBs of memory with 32 threads on a typical shared-memory machine, whereas the leading method parallel-dc3 requires 119 s using 116 GBs of memory for the sa. Our experimental study demonstrates that this superior performance of caps-sa can largely be attributed to two causes. First, caps-sa achieves better memory-locality (fewer cache misses) than the other methods (likely thanks to its straightforward approach), and second, real world use cases typically do not manifest properties that render the algorithm exhibit its worst-case complexity. However, our experimental results include performance on an adversarial dataset for the algorithm. Overall, our work demonstrates that as parallel resources increase combining domain-specific optimizations (i.e. lcp-informed merging) with highly-efficient general sorting strategies (i.e. samplesort [21]) can outperform more sophisticated but complex algorithms. caps-sa is implemented in C++17 and is available under an open source license at https://github.com/jamshed/CaPS-SA.

The remainder of this manuscript is organized as follows. We discuss the preliminary concepts required for a formal treatment of the algorithm as well as the most relevant prior work and the methods against which we compare caps-sa in Sec. 2. Then we discuss caps-sa in Sec. 3, and provide an analysis of its asymptotic behavior. Sec. 4 describes the experimental study for the proposed algorithm, and reports the results. We conclude with discussion on the potential of the method and prospective future directions for building on top of it.

## Preliminaries

A *string* (or *text*) $$T=a_0a_1\dots a_{n - 1}$$ is a finite ordered sequence of $$n$$ symbols drawn from a finite ordered alphabet $$\Sigma$$. $$\Sigma$$ contains a special terminator symbol $$\$$$, which terminates a string and is the smallest symbol in the ordering of $$\Sigma$$. $$T$$ denotes the length $$n$$ of $$T$$. The half-open interval $$[i, \, j]$$ is a shorthand for the closed interval $$[i \ldots j - 1]$$. $$T_i$$ denotes the $$i$$’th symbol in $$T$$. The *substring* $$T_{[i, j)}$$ of $$T$$ is the sequence of characters of $$T$$ in the half-open interval $$[i, \, j]$$. We call a substring $$T_{[i, j)}$$ with $$i = 0$$ a *prefix* of $$T$$. Likewise, a substring $$T_{[i, j)}$$ with $$j = |T|$$ is a *suffix* of $$T$$, denoted by $$T_{[i:]}$$.

The ordering of $\Sigma$ induces a lexicographical ordering of all possible strings over $\Sigma$. The *Suffix Array* (sa) of a string $T$ is an array of the starting indices of all suffixes of $T$ ordered by the suffixes’ lexicographical order. The *Longest Common Prefix* lcp($T_1$, $T_2$) of two strings $T_1$ and $T_2$ is the largest-sized prefix $P$ of both $T_1$ and $T_2$, such that if $|P|=k$ then for all $0<i<k$, ${T_1}_i = {T_2}_i$, and ${T_1}_k \ne {T_2}_k$. Given the suffix array $SA$ of a string $T$, its $LCP$-array is the array $L$ such that $L_i = {\textsc {LCP}}(T_{[SA_i:]}, T_{[SA_{i - 1}:]})$. 1 For instance, given the string $T=AACTGCGGAT$ the sa and $LCP$ array are given by following data structure:

**Table:** 

Index	0	1	2	3	4	5	6	7	8	9	10
$$T$$	A	A	C	T	G	C	G	G	A	T	$
$$SA$$	10	0	1	8	5	2	7	4	6	9	3
$$LCP$$ array	0	0	1	1	0	1	0	1	1	0	1

The *work* of an algorithm is the total number of operations it performs to compute the result. The *depth* (or span) of an algorithm is the longest sequence of dependent computations in its execution. In pseudo-code, we will use (||) as an infix operator to specify the parallel execution of statements—so $f(x)\ ||\ g(y)$ denotes the parallel execution of $f(x)$ and $g(y)$. We use $\mathcal {O}\big (f(n) \big )$ *with high probability (whp)* in $n$ to mean $\mathcal {O}\big (cf(n) \big )$ with probability at least $1-n^{-c}$ for some constant $c \ge 1$.

*Prior Work.* The sa can be constructed naively in $\mathcal {O}(n^2\log n)$ work for an $n$-length text. Efficient algorithms can operate in $\mathcal {O}(n)$ work and $\mathcal {O}(n)$ space, which is the theoretical optimum, as it is the time and space required to record the sa and $LCP$ array themselves. A comprehensive discussion of work on sa construction is well beyond the scope of this manuscript. As such, we here focus on several sequential and parallel algorithms which are of particular interest due to their speed and wide use.

The state-of-the-art sequential program for sa construction is divsufsort [18, 42]. Subsequent work has elucidated the algorithm to be an efficient implementation of some two-stage algorithms [18, 25].

divsufsort has been parallelized by Labeit et. al. in 2017 [37]. It has recently been used in several computational genomics tools, including the CAMMiQ method for microbial abundance quantification [56] and the macle tool for computing match complexity [47].

Another well-known algorithm for sa construction is the Difference Cover modulo 3 (DC3) method [31], which has also been effectively parallelized [36] and has a state-of-the-art implementation [3].

*Relevant String Sorting Methods.* String sorting is a well-studied algorithmic problem. The main difficulty in string sorting is that comparing two strings $T_1$ and $T_2$ requires $\mathcal {O}(\min (|T_1|,|T_2|))$ comparisons, which renders many traditional sorting algorithms for atomic objects costly. Of particular relevance to our work is the problem of *merging* two sorted lists of strings. Farach-Colton used an efficient merge-routine for building suffix trees in linear time [17] (though the space overhead of suffix trees renders them impractical for most modern applications). Ng and Kakehi analyzed an efficient merging strategy of sorted lists of strings with associated lcp-information [43]. They show that given random strings with uniform distribution of symbols, an lcp-informed merge-sort algorithm has an expected running time of $\mathcal {O}(n \log n)$ to sort $n$ strings. The same merge procedure was used by Bingmann and Sanders in several samplesorting algorithms for sorting collections of strings [8].

Bingmann and Sanders propose two merge-based string sorting algorithms of particular interest to us here: Parallel Super Scalar String Sample Sort ($pS^5$) and Parallel Multiway LCP-Mergesort. $pS^5$ makes use of the merge routine in a samplesort framework, much like caps-sa. The algorithms differ in their inputs (a set of strings vs a single string) as well as their approach to partitioning the data. $pS^5$ uses machine-word-sized pivot keys to create a binary search tree which can fit into the cache of each core, then divides up the input set of strings evenly across the cores and bins them accordingly. As described in Sect. 3.1, caps-sa divides up the input into evenly sized partitions, then samples pivots using a two-step process and places them into each partition. Parallel Multiway LCP-Mergesort generalizes the merge-algorithm to $k$-way merges [7].

## Methods

The proposed algorithm, caps-sa, is based on the *samplesort* [21] algorithm. Samplesort is a popular generalization of quicksort that achieves excellent performance on both shared-memory and distributed-memory architectures [4, 49]. Instead of partitioning the input array into two parts around a single pivot as in quicksort, it chooses a number of pivots $z_1, z_2, \ldots , z_{p - 1}$ along with two sentinel pivots $z_0 = -\infty$ and $z_p = +\infty$, and partitions the data into $p$ partitions such that an input element $x_i$ is assigned to partition $j$ iff $z_{j - 1} < x_i \le z_j$. It then sorts each partition using another (usually sequential) sorting algorithm (e.g., quicksort).

For constructing a suffix array, simply applying samplesort is costly since string comparisons in general require super-constant time. In more detail, first each suffix needs to be assigned to its partition by binary searching over the pivots. Secondly, sorting the suffixes in each partition may cost substantially more than linearithmic time due to string comparisons.

caps-sa addresses these issues using the following key idea of *jointly leveraging merge sort and* $LCP$-*arrays*. Whenever two suffixes are compared, the comparison is always done inside the operation of merging two sorted arrays of suffixes. Each sorted array is augmented with its lcp-array, and the merge operations avoid repeated comparisons of common prefixes among suffixes by exploiting these lcp-arrays. This approach has previously been used in general string sorting algorithms [7, 8, 43] and merging-based Burrows Wheeler Transform construction algorithms [11, 15, 16]. The partitioning strategy for the suffixes is modified to make better use of the merge operation and achieve good parallelism. In particular, instead of randomly sampling pivots at the beginning of the algorithm, caps-sa partitions the suffixes uniformly into $p$ subarrays, sorts the subarrays locally, and only then selects the pivots using oversampling. Once pivots are placed within each partition, the $p$ partitions are further subdivided into $p-1$ subarrays each, for a total of $p(p-1)$ sub-subarrays. Since each sub-subarray is flanked by two pivots, the partition that it should go to is known. Each partition is thus a collection of sorted sub-subarrays, which can be merged efficiently. The initial sorting of the uniform-sized subarrays is done using merge-sort to exploit the merge operation. Thus caps-sa ends up exploiting an efficient merging procedure with associated lcp-information to reduce expensive comparisons of suffixes, while not having to merge large sub-arrays due to its pivoting strategy. We discuss the algorithm in more detail in the following sections.

### Parallel SA and LCP-array construction: caps-sa

**Fig. 1:**
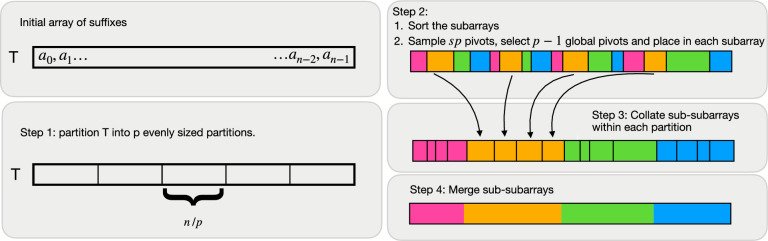
Overview of caps-sa. In the first step of the algorithm the input text $T$ is partition evenly across $p$ partitions. Then each partition is sorted, pivots are sampled using the sampling routine, and located within each partition to create sub-partitions. Subsequently each sub-partition is collated. Finally the merge routine is used to complete the suffix and the LCP-array construction


Next, we provide a high-level overview of the ${\textsc {CaPS-SA}}(T, p)$ algorithm. The input to the algorithm is a string $T$ and a partition count (or, *subproblem count*) $p$, and as output it produces the sa and the lcp-array of $T$. Conceptually, the algorithm executes in four high-level steps which we illustrate in Fig. 1.

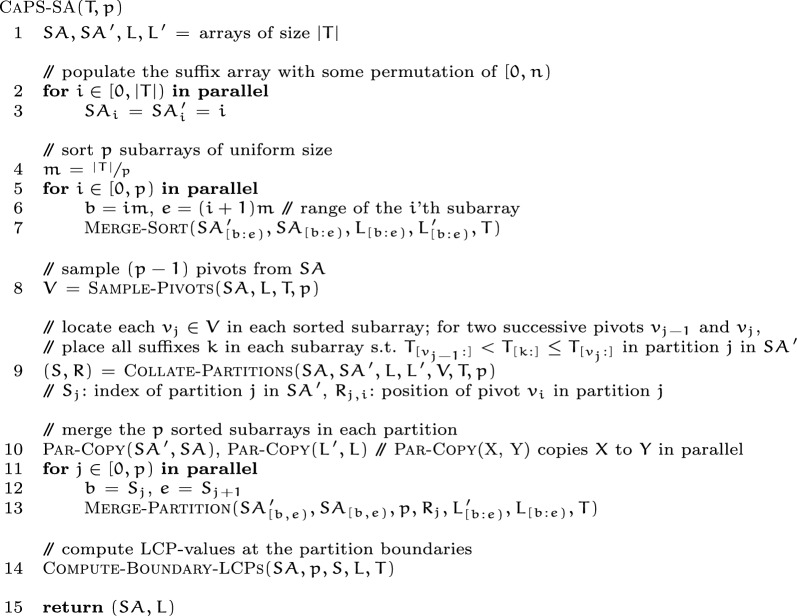


First, it populates an unsorted sa. Then this initial sa is broken into $p$ subarrays of uniform size $|T|/p$, and each subarray is sorted with merge-sort, in parallel. Next, $p-1$ global pivots are sampled from the sorted subarrays together. Then in each sorted subarray, in parallel, each pivot is located with a binary search. The locations of the $p - 1$ pivots thus found in each sorted subarray break the subarray into *p* sorted 
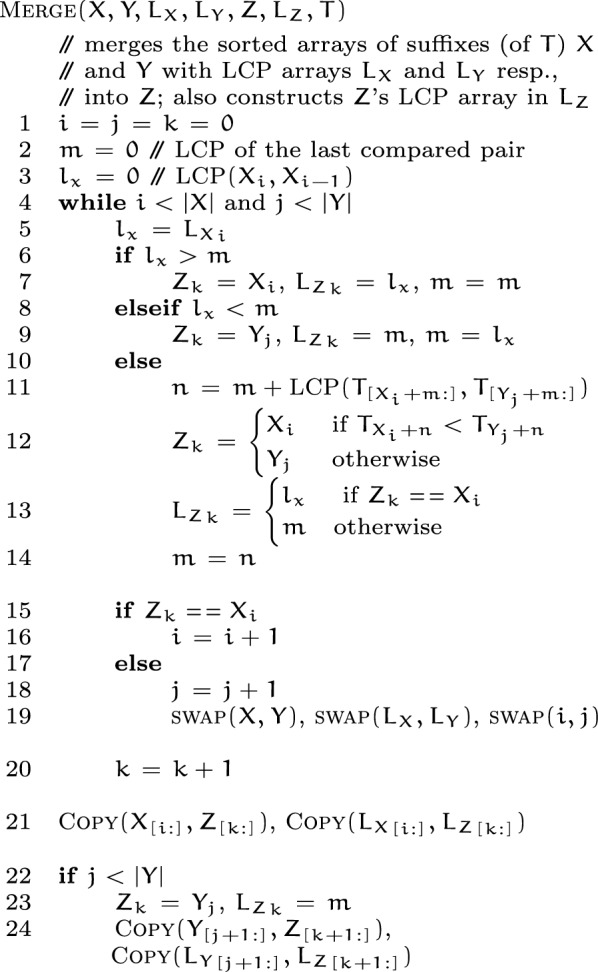

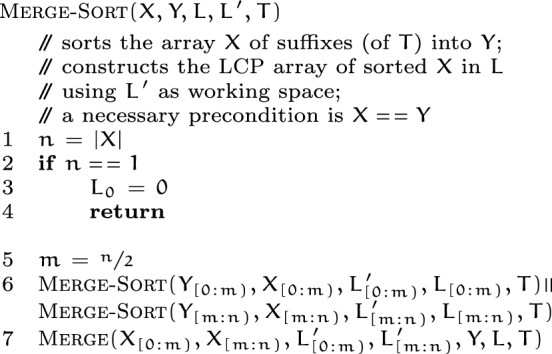

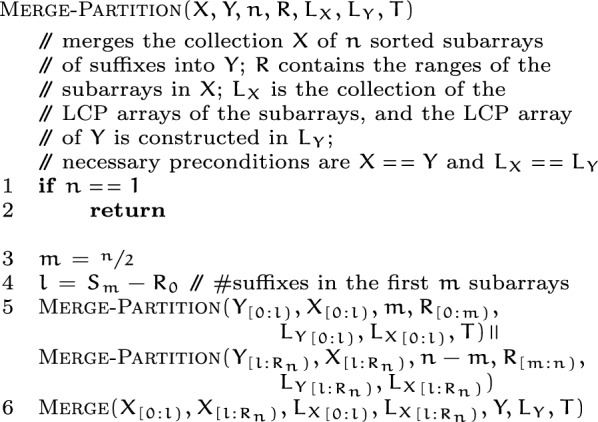
 sub-subarrays. Besides, the position of each pivot in the final sa is now defined by its location in each of the $p$ subarrays. The local ordering of the suffixes in each sorted subarray and the global position of the pivots thus define $p$ partitions for the final sa, each of which is a collection of $p$ sub-subarrays: one from each of the $p$ sorted subarrays. Then for each partition, in parallel, its $p$ sorted sub-subarrays are merged recursively into a fully sorted partition. Together, these sorted partitions, in order, produce the final sa and the final lcp-array. The lcp-values for pairs that cross partition boundaries are computed at the end.

The algorithm is presented as following, and its major steps are detailed in the following subsections. Then we analyze the asymptotic characteristics of the algorithm.

The merge operation. For efficient suffix comparisons, caps-sa utilizes the merge operation. A pair of suffixes is compared only when merging two sorted lists of suffixes, with the only exception being the case when the algorithm performs a binary search using a pivot suffix. When merging sorted suffixes, merging without any extra information about the suffixes in its input lists can be costly due to super-constant time string comparisons. To avoid comparing repeated prefixes of suffixes, the merge procedure in caps-sa utilizes the lcp-arrays of the input suffix lists, generated recursively in the merge-sort procedure.

The ${\textsc {Merge}}(X, Y, L_X, L_Y, Z, L_Z, T)$ procedure takes two sorted arrays $X$ and $Y$ of suffixes, their respective lcp-arrays $L_X$ and $L_Y$, and populates the array $Z$ as the merged output for $X$ and $Y$. Also, the lcp-array of $Z$ is produced in $L_Z$. The procedure works exactly like the classic merge routine, with the following modifications.

**Fig. 2:**
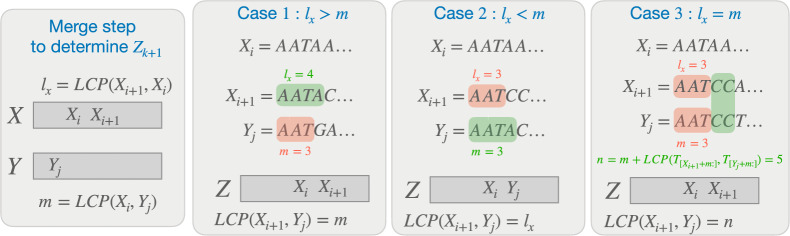
Figure illustrating the cases that can occur on the $(k+1)$’th step of the merge routine, which determines $Z_{k+1}$. Cases 1 and 2 require $\mathcal {O}(1)$ work and simply compare the LCP-lengths of the previous step and ${\textsc {LCP}}{}(X_{i+1}, X_{i})$, which are already available. Step 3 requires work proportional to $\mathcal {O}({\textsc {LCP}}(X_{i+1}, Y_{j}) - m)$, since $X_{i+1}, Y_{j}$ already share a prefix of size $m$

At a given moment, let $X_i$ and $Y_j$ be the two suffixes being compared, and $Z_k$ be the output of the comparison. Without loss of generality, say that $X_i < Y_j$ is found, i.e. $Z_k = X_i$. Let $m$ denote the lcp-length of the the last compared pair in each step of the merge. Then after the current step finishes comparing $X_i, Y_j$, we have that $m = {\textsc {LCP}}(X_i, Y_j)$. $X_i < Y_j$ implies that $T_{X_i + m} < T_{Y_j + m}$. The next suffixes to compare are $X_{i + 1}$ and $Y_j$. Let $l_x = {L_X}_{i + 1} = {\textsc {LCP}}(X_{i + 1}, X_i)$. $X_{i + 1} > X_i$ implies that $T_{X_{i + 1} + l_x} > T_{X_i + l_x}$. There are three possible outcomes when comparing $l_x$ and $m$ (illustrated in Fig. 2):

1. $l_x > m$: It implies that $T_{X_{i + 1} + m} = T_{X_i + m}$. Combining with $T_{X_i + m} < T_{Y_j + m}$, we get $T_{X_{i + 1} + m} < T_{Y_j + m}$. It follows that
  $$\begin{aligned} Z_{k + 1} = X_{i + 1}, {L_Z}_{k + 1} = {\textsc {LCP}}(Z_{k + 1}, Z_k) = {\textsc {LCP}}(X_{i + 1}, X_i) = l_x, m = {\textsc {LCP}}(X_{i + 1}, Y_j) \! = m \end{aligned}$$
2. $l_x < m$: It implies that $T_{X_i + l_x} = T_{Y_j + l_x}$. Combining with $T_{X_{i + 1} + l_x} > T_{X_i + l_x}$, we get $T_{X_{i + 1} + l_x} > T_{Y_j + l_x}$. It follows that
  $$\begin{aligned} Z_{k + 1} = Y_j, {L_Z}_{k + 1} = {\textsc {LCP}}(Z_{k + 1}, Z_k) = {\textsc {LCP}}(Y_j, X_i) = m, m = {\textsc {LCP}}(X_{i + 1}, Y_j) = l_x \end{aligned}$$
3. $l_x {==}m$: We compute $n = m + {\textsc {LCP}}(T_{[X_{i + 1} + m:]}, T_{[Y_j + m:]})$, and set the following:
  $$\begin{aligned} Z_{k + 1} = {\left\{ \begin{array}{ll} X_{i + 1} &{} \text {if } T_{X_{i + 1} + n} < T_{Y_j + n}\\ Y_j &{} \text {otherwise} \end{array}\right. } \end{aligned}$$
  $$\begin{aligned} {L_Z}_{k + 1} = {\textsc {LCP}}(Z_{k + 1}, Z_k) = {\left\{ \begin{array}{ll} {\textsc {LCP}}(X_{i + 1}, X_i) = l_x &{} \text {if } Z_{k + 1} {==}X_{i + 1} \\ {\textsc {LCP}}(Y_j, X_i) = m &{} \text {otherwise} \end{array}\right. } \end{aligned}$$
  $$\begin{aligned} m = {\textsc {LCP}}(X_{i + 1}, Y_j) = n \end{aligned}$$

The merge procedure continues this way through $X$ and $Y$. Finally, when either of $X$ and $Y$ has been depleted, the rest of the entries at the other one are copied to the end of $Z$ and $L_Z$.

*Local sorting.* caps-sa starts out with some permutation of $[0,\, |T|)$, and sorts its $p$ disjoint subarrays, each of size $|T|/p$, in parallel using merge-sort. The ${\textsc {Merge-Sort}}(X, Y, L, L', T)$ procedure takes as input an array $X$ of suffixes, and sorts it into $Y$. Besides, the lcp-array of sorted suffixes is produced in $L$, using $L'$ as working space. As typical merge-sort implementation requires linear extra space in each invocation, caps-sa uses the arrays $X$ and $Y$ in a back-and-forth manner to reuse the extra space in the invocations. For such, $Y$ needs to be equal to $X$ before an invocation. The merge step in the sort uses the merge-procedure described earlier.

*Pivot selection.* caps-sa deviates from samplesort in its pivot selection strategy. In a typical samplesort, pivots are to be sampled from the initial array and then partitioning would be based on their intervals. Instead, in parallel, the ${\textsc {Sample-Pivots}}(SA, T, p)$ procedure (see Suppl.) in caps-sa samples $s$ suffixes from each of the $p$ subarrays, where $s$ is the *sampling factor*. Then these $s\times p$ sample suffixes are sorted using merge-sort. Subsequently, $p - 1$ evenly-spaced pivots are selected from the sorted output to form the pivot set $V$.

These pivots define the ranges of the samplesort partitions, and are used to split each of the subarrays in the next collation step. We show in Theorem 1 that with a sufficient sampling factor $s$, the size of each partition is within a constant factor of $|T|/p$ with high probability, which ensures a balanced load for processing each partition in the last step of the algorithm.

*Collating partitions.* Having finalized the pivot set $V$, the algorithm locates each pivot suffix $v \in V$ in each sorted subarray. Each subarray is searched for the $p - 1$ pivots in parallel.

Consider a pivot $v \in V$ and some sorted subarray $A$. The position of $v$ in $A$ is the last index where $v$ can be inserted without breaking the sorted order of $A$. This index is computed using a binary search for the suffix $v$ in $A$. During a binary search suffixes are compared without any associated $LCP$ array, contrary to the merge procedure. As a practical speedup, we skip some repeated character comparisons between $v$ and the suffixes in $A$ using the *simple accelerant* idea [22].

After placing each pivot $$v$$ into $$A$$, the index $$i$$ of $$v$$ in $$A$$ implies that all the suffixes in $$A_{[0: i)}$$ are $$\le v$$. Hence the sum $$C_v$$ of these indices of $$v$$ across all the $$p$$ sorted subarrays provides the total suffix count in the sa that are not lexicographically larger than $$v$$—the index of $$v$$ in the final sa is $$C_v - 1$$. Along with the sentinel pivot positions $$C_0 = 0$$ and $$C_p = |T|$$, these $$p - 1$$ pivots divide the final sa into $$p$$ partitions. Consider two successive pivots $$v_{j - 1}$$ and $$v_j$$. In each sorted subarray $$A$$, all the suffixes $$k$$ such that $$T_{[v_{j - 1}:]} < T_{[k:]} \le T_{[v_j:]}$$ will be present in the index-range $$[C_{j - 1}, C_j)$$ of the final sa. That is, all the suffixes between the locations for $$v_{j - 1}$$ and $$v_j$$ belong to the $$(j - 1)$$’th partition.

Thus the pivot locations in a sorted subarray $A$ break $A$ into $p$ sub-subarrays, where the $j$’th sub-subarray is known to be present in the $j$’th partition of the final sa. After the binary searches, caps-sa moves these sub-subarrays in parallel to collate all sub-subarrays for the same partition. The lcp-arrays of these sub-subarrays are also collated together. The ${\textsc {Collate-Partitions}}(SA, SA', L, L', V, T, p)$ procedure (see Suppl.) describes it in more detail.

*Merging partitions.* Having grouped together the corresponding sub-subarrays for every partition, caps-sa merges together the sorted sub-subarrays in each partition, in parallel. A partition consists of $p$ sorted collections of suffixes, with all of the collections stored contiguously. The ${\textsc {Merge-Partition}}(X, Y, n, R, L_X, L_Y, T)$ procedure takes this collection $X$ of $p$ sorted sub-subarrays, and produces the merged output in the same contiguous region of memory $Y$ recursively. $L_X$ is the collection of the lcp-arrays of the sorted groups in $X$, and the merged lcp-array is produced in $L_Y$. The sorted groups in $X$ (and $L_X$) are delineated by $R$.

The merge-partition procedure is same as the merge-sort procedure, except for that it is more general—the sorted units where merge-sort bottoms out are single suffixes, whereas merge-partition bottoms out earlier at sorted groups of suffixes. As noted earlier, merge-partition also uses the space in $X$ and $Y$ back-and-forth to reuse the extra spaces required.

### Asymptotics

In this section, we analyze the computational complexity of the ${\textsc {CaPS-SA}}(T, p)$ algorithm executed on a text $T$ with length $n = |T|$, given a subproblem-count $p$.

#### Work analysis

We start by analyzing the overall work of the algorithm and providing self-contained proofs on the total work due to symbol comparisons made by our algorithm.

*Local sorting.* This step executes the classic merge-sort on each subarray. For a subarray $A$ with $m$ suffixes, this amounts to a total work of $T(m) = 2 T({m}/{2}) + \mathcal {O}(m) + C(A)$, where $C(A)$ denotes the number of symbol comparisons made in the execution in the third case of the merge procedure. We analyze the total amortized cost of these $C(A)$ values across all the recursion-trees of all the subarrays in Theorem 3. Omitting $C(A)$ from $T(m)$, each local sort has ${n}/{p}\log {{n}/{p}}$ work.

*Pivot selection.* With a sampling factor $s$, there are $s\times p$ pivots sampled in total across all the subarrays. caps-sa sorts these pivots with merge-sort and picks the $p - 1$ equidistant pivots from these as the global pivots. The merge-sort amounts to a total work of $\mathcal {O}(sp \log {sp} + \sum {L_p}_i)$, where $L_p$ is the output lcp-array of the sort. This holds from Theorem 3.

*Collating partitions.* The collation step first locates each of the $p - 1$ pivots in each of the sorted subarrays using binary search. The length of a pivot suffix is $\mathcal {O}(n)$, and the sorted subarrays are of size ${n}/{p}$. The work of each binary search is $\mathcal {O}(n + \log {{n}/{p}})$ in practice ($n = |T|$) with the simple-accelerant [22] strategy. For adversarial inputs however, the work can still be $\mathcal {O}(n \log {{n}/{p}})$ in the worst-case. Then the suffix indices are moved into their appropriate final partitions. This step simply reorders the elements across the sorted subarrays, and thus requires $\mathcal {O}(n)$ total work.

*Merging partitions.* The merge-partition procedure works similar to the merge-sort procedure, except for that the recursion bottoms out at a sorted group of suffixes, instead of at a single suffix. Unlike the merge-sort instances however, each of which operate on ${n}/{p}$-sized subarrays, the merge-partition instances may work on various sizes of partitions. Theorem 1 provides a bound on the partition sizes.

##### Theorem 1

With a sampling factor $s$, every partition has size at most ${cn}/{p}$ for some constant $c$ with high probability.

##### Proof

The algorithm samples $s$ pivots from each subarray, for a total of $sp$ samples.

It then picks $p - 1$ evenly spaced pivots (every $s$’th sample) from the sorted samples to use as global pivots.

Consider the final location of these $sp$ samples in the final suffix array.

Every $s$’th of them marks the boundary of a partition.

Thus, a partition has size $d \ge {cn}/{p}$ only if fewer than $s$ of the samples fall into these $d$ suffixes.

Otherwise, at least one sample would be picked as a final pivot and would thus break this partition.

Let $SA$ be the final suffix array, and $X_i$ be a random variable indicating whether ${SA}_i$ is one of the $sp$ samples.

Then $$Pr[X_i = 1] = {sp}/{n}$$.

Thus the random variable denoting the number of samples picked from a region of size ${cn}/{p}$ is $X = \sum _{i = 1}^{{cn}/{p}} X_i$. By linearity of expectation, we get $E[X] = \sum _{i = 1}^{{cn}/{p}} E[X_i] = {cn}/{p} Pr[X_i = 1] = {cn}/{p} \cdot {sp}/{n} = cs$. Applying the Chernoff bound we have:

1 $$\begin{aligned} Pr[X < s] \le Pr[X \le s] &= Pr[X \le \frac{1}{c}E[X]] \\ & = Pr[X \le (1 - (1 - \frac{1}{c}))E[X]] \\ & \le \exp ( - 1/2(1 - 1/c)^{2} E[X]) \\ & = \exp ( - 1/2(1 - 1/c)^{2} cs) \\ \end{aligned}$$

With $s = 32 \ln n$ and letting $c' = c (1 - {1}/{c})^2$,

$$\begin{aligned} Pr[X < s] \le \exp \big (-{1}/{2} (1 - {1}/{c})^2 c \cdot 32 \ln {n} \big ) = \exp \big (\ln (n^{-16 c (1 - {1}/{c})^2}) \big ) = {1}/{n^{16c'}} \end{aligned}$$

Since the event of a partition having size $\ge {cn}/{p}$ implies the event $X < s$, we get:

$$Pr[\text {a given partition has size} \ge {cn}/{p}] \le Pr[X < s] \le {1}/{n^{16c'}}$$.

$\Rightarrow Pr[\text {at least one partition has size } \ge {cn}/{p}] \le \sum _{i = 1}^p {1}/{n^{16c'}} = {p}/{n^{16c'}}$ (by union-bound).

$$\Rightarrow Pr[\text {no partition has size} \ge {cn}/{p}] \ge 1 - {p}/{n^{16c'}}$$.

Since $p$ is at most $\mathcal {O}(n)$, no partition has size $\ge {cn}/{p}$ *whp*. $\square$

Thus each partition has size at most ${cn}/{p}$ for some constant $c$ *whp*. Merging a partition $A$ with $m$ sorted subgroups has work $T(m) = 2T({m}/{2}) + \mathcal {O}(m) + C(A)$, where $C(A)$ is the number of symbol comparisons made in merge. Omitting $C(A)$ from $T(m)$, this solves to $\mathcal {O}({cn}/{p} \log {p}) = \mathcal {O}({n}/{p}\log {p})$ *whp*.

*Total symbol comparisons.* A subproblem in the algorithm is some subarray of the sa that can be processed independently of the other subarrays in a given step. Let $X$ be some subproblem in either of the two steps: local-sorting and partition-merging. For the local-sorting case, sorting $X$ with merge-sort consists of $\log {n}/{p}$ recursion-levels. For the partition-merging case, the merge-partition procedure for $X$ executes in $\log {p}$ recursion-levels. We label the bottom-most level as level 0, and count the levels upwards in the recursion-tree.

Let $x \in X$ be a suffix. At any given level $i, x$ is present in exactly one merge-sort (or merge-partition) instance executing on $X$. Let $x'_i$ be the suffix that immediately precedes $x$ in the output of that merge-sort (or merge-partition) instance, and let $L_i(x) = {\textsc {LCP}}(x, x'_i)$. If $x$ is the first suffix in the output, then $x'_i$ is the empty suffix. We prove the following.

##### Theorem 2

In merge-sort and merge-partition, $L_i (x) \le L_{i + 1}(x)$ for a suffix $x$ at each recursion level $i \in [0, d - 1)$, where $d$ is the depth of the recursion-tree.

##### Proof

Let $x$ be present in the merge-sort (or merge-partition) instance $M$ at level $i + 1$, and say $M$ spawns the two instances $M_l$ and $M_r$. $M_l$ and $M_r$ are at level $i$, and $x$ is present in exactly one of them. Let it be $M_l$.

Now, $x'_{i + 1}$ is either $x'_i$ i.e. the same suffix preceding $x$ in $M_l$, or some other suffix $y$ from $M_r$. If $x'_{i + 1} = x_i$, then $L_{i + 1}(x) = L_i(x)$, and the claim holds.

In the other case, the output array of $M$ has the following form: $[\ldots , x'_i, \ldots , y, x, \ldots ]$. Suppose that the claim is false, i.e. $L_i(x) > L_{i + 1}(x)$. Which is, ${\textsc {LCP}}(x, x'_i) > {\textsc {LCP}}(x, y)$. ${\textsc {LCP}}(x, y) < {\textsc {LCP}}(x, x'_i)$ implies that $x'_i$ and $y$ share the same prefix of length $l = {\textsc {LCP}}(x, y)$, and mismatch first at index $l$. Let $c_x, c_{x'_i}$, and $c_y$ be the $l$’th symbol in $x$, $x'_i$, and $y$ resp. As $y > x'_i$ in the output, $c_y > c_{x'_i}$. Besides, since $x$ and $y$ first mismatch at the $l$’th symbol and $x > y$, $c_x > c_y$. Thus $c_x > c_{x'_i}$. ${\textsc {LCP}}(x, x'_i) > l$ implies that the $l$’th symbols in $x$ and $x'_i$ are the same, i.e. $c_x = c_{x'_i}$. Thus we get $c_x > c_{x'_i}$ and $c_x = c_{x'_i}$, resulting in a contradiction. Hence, ${\textsc {LCP}}(x, y) \le {\textsc {LCP}}(x, x'_i)$. $\square$

Theorem 3 provides a bound on the number of total comparisons made across all the merge-sort and merge-partition instances in the algorithm execution.

##### Theorem 3

The total number of symbol comparisons made across all the merge-sort and merge-partition instances in caps-sa for an $n$-length text is $\mathcal {O}( n \log {n} + \sum _{i = 1}^{n - 1} L_i \big )$ *whp*, where $L$ is the output lcp-array.

##### Proof

Symbol comparisons occur only as part of the merge procedure in both merge-sort and merge-partition. Given two sorted lists of suffixes $X$ and $Y$ along with their lcp-arrays, the ${\textsc {Merge}}(X, Y, L_X, L_Y, Z, L_Z, T)$ procedure iterates through the $X_i$’s and $Y_j$’s and fills in the $Z_k$’s in the sorted order, along with their lcp-values in ${L_Z}_k$. Without loss of generality, suppose that $X_i < Y_j$ is found at some iteration. Then the next iteration compares $X_{i + 1}$ and $Y$. Let $l_x = {\textsc {LCP}}(X_{i + 1}, X_i)$ and $m = {\textsc {LCP}}(X_i, Y_j)$, lcp of the last compared pair. Symbols from the suffixes $X_{i + 1}$ and $Y_j$ will only be compared iff $l_x = m$ holds. In this case, we compute $n = {\textsc {LCP}}(X_{i + 1}, Y_j)$ with exactly $n - m + 1$ symbol comparisons. The $+1$ term is due to the first mismatching symbol pair. $m$ is set to $n$ for the next iteration. We argue that before the new $m = n$ value is assigned as the lcp-value in the output lcp-array $L_Z$ in some future iteration, it remains unchanged.

In the next iteration, if case (1), i.e. $l_x > m$ holds, then $m$ remains unchanged. If case (2), i.e. $l_x < m$ holds, then $m$ is assigned at output ${L_Z}_{k + 1}$. In the event of case (3), either $l_x$ or $m$ is assigned to ${L_Z}_{k + 1}$, and these are equal in this case. If $X$ has been depleted during the merge while $Y$ still has remaining elements, the current $m$ is assigned as the lcp-value for the first of the remaining elements from $Y$.

Thus, whenever symbol comparisons are done in case (3) of merge, it results in a new value $m' \ge m$ for the variable $m$. $m = m'$ persists until $m'$ has been assigned as the lcp-value for some merged output. Thus the number of matching symbol comparisons made in case (3) accumulates in the lcp-values at the output.

All the lcp-values start out with $0$ at merge-sort. Theorem 2 states that the lcp-value associated to a given suffix can never decrease while winding up the recursion-trees of merge-sort and merge-partition. Thus the sum $\sum _{i = 1}^{n - 1} L_i$ of the final lcp-values in the sa is the total number of matching symbol comparisons made across all the merge-sort and merge-partition executions.

The extra mismatching comparison in case (3) of merge costs $\mathcal {O}(1)$. In the worst case, this case occurs in each iteration of merge. Omitting the matching symbol comparisons, a merge-sort or a merge-partition instance working on $m$ elements incurs $T(m) = 2T({m}/{2}) + \mathcal {O}(m)$ mismatches in the worst case. This solves to $p \times \mathcal {O}({n}/{p} \log {{n}/{p}})$ and $p \times \mathcal {O}({n}/{p} \log {p})$ *whp* for the $p$ merge-sorts and merge-partitions, resp. Thus $\mathcal {O}(n\log {n})$ mismatching symbol comparisons are made *whp*. $\square$

*Total work.* Locally sorting the $p$ subarrays cost $p \times \mathcal {O}({n}/{p} \log {{n}/{p}}) = \mathcal {O}(n\log {{n}/{p}})$ work without the symbol comparisons. Omitting the symbol comparisons in sorting the sampled pivots, the pivot selection step has $\mathcal {O}(sp \log {sp})$ work. In the collation step, there are $p(p - 1)$ binary searches, costing $\mathcal {O}(p^2 n\log {{n}/{p}})$ work in the worst-case, and $\mathcal {O}(p^2 \big (n + \log {{n}/{p}}) \big )$ in practice. Merging the $p$ partitions separately cost $p \times \mathcal {O}({n}/{p}\log {p}) = \mathcal {O}(n\log {p})$ *whp* without the symbol comparisons.

The total number of symbol comparisons in the local-sort and the partitions-merge steps is $\mathcal {O}(n \log n + \sum _{i = 1}^{n - 1} L_i)$ *whp* as per Theorem 3, where $L$ is the output lcp-array. In sorting the sampled suffixes, the number of symbol comparisons done is also bounded by this 2. Thus the total work for the algorithm is $\mathcal {O}(n \log n + \sum _{i = 1}^{n - 1} L_i)$ *whp*. 3

### Working space

The working space of the algorithm is the total space required by all the merge procedure instances at any given recursive-level of merge-sort or merge-partition. The merge procedure produces two merged output for some given input, the sorted suffixes and their LCPs, and thus has $2 \times 2|T|$ input and output entries in total at any level. So the working space for the algorithm is $4n$ entries, which is $\mathcal {O}(n\log {n})$ bits. Our implementation of the algorithm requires $4w|T|$ bytes of working space, where $w\in \{4,8\}$ is the numerical size used to store sa and lcp values. We discuss an external-memory scheme to reduce the working space in Sec. 5.

### Parallelization

Our implementation fully parallelizes the work across the different partitions. Within a partition, we perform recursive calls to merge-sort in parallel, but perform the merge procedure serially. We show the following theorem about the depth of our algorithm:

#### Theorem 4

The overall depth of the algorithm is $\mathcal {O}\big ((n/p) \log n \big )$ *whp*.

#### Proof

The dominant factor in the merge algorithm is the depth of the merge routine, which simply performs a linear number of comparisons in the input size. The depth of a comparison is $\mathcal {O}(1)$ in cases 1 and 2 of Fig. 2, and requires a string comparison in the final case.

The string comparison can be parallelized work-efficiently (i.e., in the same work as a serial character-by-character comparison) by using a simple prefix-doubling strategy. In more detail, the comparison algorithm works in rounds comparing $2^i$ characters in the $i$’th round until a mismatch occurs. Clearly for strings of length $\mathcal {O}(n)$ only $\mathcal {O}(\log n)$ rounds are required, and thus the overall work is asymptotically the same as the serial algorithm, and the depth is $\mathcal {O}(\log n)$. Thus, for merging two sorted arrays in the algorithm, each of size $k$, we require a depth of $\mathcal {O}(k \log n)$.

Putting these facts together, for a single call to the merge-sort routine, we have a recurrence of the form $D(k) = D(k/2) + \mathcal {O}(k \log n)$, which is root dominated and solves to $\mathcal {O}(k\log n)$. Since our algorithm is parallelized across different partitions, and by Theorem 1 each partition has size at most $\mathcal {O}(n/p)$, the overall depth of the algorithm is $\mathcal {O}((n/p) \log n)$ *whp*. $\square$

We note that the depth is not poly-logarithmic, as in the classic parallel merge-sort. However, the amount of parallelism generated by our algorithm is more than enough to keep the processors all busy in practice. Indeed, we note that many samplesort implementations use a similar strategy in practice and use a serial sort within each partition, and thus also do not have poly-logarithmic depth in practice. In our implementation, we exploit parallelism using the parallel primitives and the work-stealing scheduler from ParlayLib [10].

### Optimizations

We applied a number of optimizations into the implementation of the algorithm that provide practical speedups. We make use of vectorization support using AVX instructions from modern processors to speed up the computation of the ${\textsc {LCP}}(X, Y)$ routine used in the merge-procedure and in the binary searches in locating pivots.

In the proposed merge-sort and the merge-partition procedures in the algorithm, we have nested parallelism for their recursive invocations. This is applied in the implementation up-to some fixed granularity, due to the associated overhead of scheduling small tasks.

In the binary searches for the sampled pivots in each sorted subarray, instead of searching for the appropriate position of an entire pivot suffix, we look for a fixed-length prefix of the pivot. This helps reduce the total work associated to locating the pivots, with an associated trade-off with the final partition sizes. With sufficiently large prefix lengths, the partition sizes do not get significantly affected in our observation.

## Results

We performed a number of experiments to characterize the performance of the caps-sa algorithm and its implementation. We evaluated its performance compared to the available implementations of two leading methods for sa construction: parallel-divsufsort [37] and parallel-dc3 [3]. We assessed its ability to construct sa and lcp-arrays on a number of genomic datasets.

Next, we evaluated the parallel scaling of the algorithm. Then we explore the idea of *Bounded-context suffix arrays*, and the performance of caps-sa for various prefix-context lengths.

A varied collection of datasets has been used in the experiments. Table 1 delineates the pertinent characteristics of the datasets. We follow [51] by removing N-repeats, which occur when the sequence underlying a region of the assembly cannot be resolved. We also un-mask soft-masked regions of the genomes. We verified the correctness of the implementation by cross-checking its output against from that of parallel-divsufsort.

*Computation system.* The experiments have been performed on a server having 4 Intel(R) Xeon(R) Platinum 8160 processors with 192 cores in total and 1.5 TB of 2.66 GHz DDR4 RAM. The system is run with Ubuntu 22.04.2 LTS (GNU/Linux 6.2.0–33-generic x86_64). The sa and lcp-array construction times and the maximum memory usages of the tools were measured with the GNU time command.

### Dataset characteristics

Table 1 provides some pertinent characteristics of the datasets used. The GRCh38 dataset is the Human Build 38 patch release 13 version of the human genome reference from the Genome Reference Consortium,^4^ which is a chromosome-level assembly of the full genome. The T2T dataset is the latest T2T CHM13v2.0 Telomere-to-Telomere assembly of the CHM13 cell line with chromosome Y from NA24385, from the T2T consortium, which is a complete genome-level assembly of the genome [45]. Together, these two human datasets represent what we imagine may be a *typical* use-case for genome construction in the context of a tool like STAR [14]. Though largely similar, the CHM13 assembly has resolved telomeric and centromeric regions, and more complete coverage, specifically in highly-repetitive regions. Thus, we expect it represents a more challenging problem instance for suffix array construction.

The CdBG (Compacted de Bruijn Graph) dataset is the collection of the maximal unitigs extracted from the de Bruijn graph (with *k*-mer size 27) of the human sequencing read set NIST HG004 (SRA3440461—95) [57] by cuttlefish 2 [32]. This dataset represents a potential use-case where one may wish to build an index for the sequence stored in the CdBG data structure. The ability to index the CdBG (i.e. contigs-based indexing) has proven useful in many contexts [12], and the sa can provide one possible index for providing efficient lookup over the sequence contained in the CdBG.

The great white shark dataset is the genome reference of *Carcharodon carcharias* [41] and the axolotl dataset is the genome reference sequence of *Ambystoma mexicanum* [52]. These represent large problem instances, where one may wish to build the sa on large reference genomes.

The bacteria (1K) dataset is a collection of 1000 genomes sampled randomly from 661,405 bacterial genomes [9], from the European Nucleotide Archive. Almost all the genomes in this dataset are *Salmonella enterica*—this represents a pathological dataset for caps-sa, being extremely repetitive.

**Table 1:** Dataset statistics: number of bases, mean LCP, and standard deviation (rounded to nearest whole number) of the final lcp-array

Dataset	Size	Mean LCP	Std. Dev. of LCP
Human (GRCh38)	2,945,849,068	3814	72,697
Human (T2T)	3,117,292,071	2519	61,987
CdBG (Human reads)	3,993,272,308	18	6
Great white shark	4,286,311,195	489	8925
Axolotl	28,203,219,824	50	160
Bacteria (1K)	3,919,109,158	10,897	28,463

### SA and LCP-array construction

We evaluate the performance of caps-sa in constructing the sa and the lcp-array of a number of genomic datasets, compared to the sa construction performance of: 1. parallel-divsufsort [37] and 2. parallel-dc3 [3]. Table 2 contains the results of the benchmarking. As the state-of-the-art sequential benchmark, we note the performance of the divsufsort implementation from libdivsufsort [42].

**Table 2:** Time- and memory-performance results for constructing sas (and lcp-array in case of caps-sa) with 32 threads

Dataset	Divsufsort	Parallel-divsufsort	Parallel-dc3	Caps-sa
Human (GRCh38)	556 (25)	273 (**32**)	113 (110)	**93** (45)
Human (T2T)	575 (26)	279 (**34**)	119 (116)	**106** (48)
CdBG (Human reads)	722 (33)	379 (**44**)	149 (172)	**114** (61)
Great white shark	771 (36)	410 (**48**)	176 (186)	**121** (64)
Axolotl	10489 (236)	3424 (**311**)	–	**1341** (848)
Bacteria (1K)	726 (33)	437 (**43**)	**164** (172)	259 (60)

divsufsort is shown as a serial benchmark. Time is reported in seconds, and the memory usages are reported in GBs in parentheses. Best performances among the parallel algorithms in each instance are highlighted. parallel-dc3 could not be run on Axolotl because we could not modify the PBBS code-base to accommodate the large numerical size

We note that caps-sa executes significantly faster than the other parallel algorithms in all the instances except for the pathological dataset of 1K similar bacteria, whereas parallel-divsufsort uses the least amount of memory. Interestingly for the smaller datasets caps-sa does not require much more memory than parallel-divsufsort, despite constructing both the sa and lcp. Memory usage could be improved by bit-packing the indexes, or through the extension to an external memory algorithm.

Appendix Table 4 provides sequential timing results for the methods, i.e. with 1 thread, to compare their total amount of work. We note that caps-sa tends to do more work than the other parallel methods—which is expected as the other methods have $\mathcal {O}(n \log n)$ work [36, 37], whereas caps-sa has an additional $\mathcal {O}(\sum _{i = 1}^{n - 1} L_i)$ output-dependent factor, and benefits from better parallelization with more workers.

### Parallel scaling

**Fig. 3:**
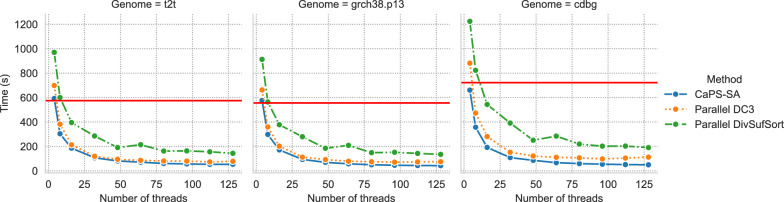
Runtimes of caps-sa, parallel-divsufsort, and parallel-dc3 as thread count increases from 4 to 128 for GRCh38, T2T, and CdBG. divsufsort runtime is in red

In order to assess how sensitive runtime is to parallelism we evaluated caps-sa against parallel-dc3 and parallel-divsufsort as the number of threads increased. We report the results in Fig. 3, which illustrated that caps-sa exploits parallelism better—becoming the fastest method as the thread count becomes high despite doing more work asymptotically.

On the GRCh38 and T2T datasets, all the parallel methods become faster than divsufsort at around the same number of threads, after which caps-sa becomes the fastest.

### Cache performance

The samplesort-based design of caps-sa optimizes cache-performance. In order to evaluate the empirical cache behavior of caps-sa as compared to other algorithms for sa construction, we profiled the programs on the GRCh38 and the T2T reference genomes. Because cache-behavior can degenerate as parallelism increases, we evaluate it across 1, 32, and 64 threads. The results in Table 3 show that caps-sa outperforms other parallel sa indexing programs by large margins. All measurements were taken with the Linux perf command.

**Table 3:** Cache-miss rates (in %) for compared methods on GRCh38 and T2T datasets with respect to number of threads

Method	Human (GRCh38)			Human (T2T)		
	1 thr	32 thr	64 thr	1 thr	32 thr	64 thr
caps-sa	**24**	**32**	**37**	**43**	**53**	**60**
parallel-divsufsort	59	60	61	60	61	62
parallel-dc3	72	68	68	72	68	68
divsufsort	51	-	-	52	-	-

Reported numbers are the minimum of 3 runs to obviate operating system jitter. Lower is better, and the best result is highlighted

### Bounded-context SA Construction

By virtue of organizing all suffixes of the underlying text $T$, the suffix array provides the powerful ability to efficiently search for query patterns of *any* length in the text. While this capability arises naturally from the definition of the sa, such flexibility is rarely needed in the sa’s most common applications in genomics. Specifically, when used to efficiently find *seed* sequences from a genomic read, the maximum length of the query is often very short. Many modern aligners use seed lengths in the range of 15–31, and even with the maximum mappable prefix concept used by STAR [14], the query length is bounded above by the error-free prefix length of the remainder of the read (rarely more than $\sim 100$ nucleotides).

As such, indices that can provide efficient lookup and locate queries for patterns less than some maximum length, say $k$, are often very useful in this context. For example, the $k$-BWT data structure [13, 46, 50] builds a transform of the text that organizes character occurrences by their *bounded context* (in this case, their right context of length $k$). This allows the index to be built efficiently, since rotations of the text need not have their relative orders resolved beyond their initial length $k$ contexts, while simultaneously allowing efficient and correct query for any pattern length $\le k$.

Here, we experiment with an analogous version of the sa—the *bounded-context* $SA$. Specifically, the bounded-context sa of order $k$ resolves the lexicographic order of all suffixes of the text up to (and including) their prefixes of length $k$. If a pair of suffixes share a prefix of length $\ge k$, then they may appear in an arbitrary relative order within the bounded-context sa of order $k$. Without any meaningful modifications to the query algorithms, this variant of the sa allows locating all occurrences of queries of any length $\le k$ in the text. Such a variant of the sa is very straightforward to construct using caps-sa, as we simply declare equal any suffixes that are equal up to (or beyond) their length $k$ prefixes. At the same time, this variant can be more efficient to construct using our algorithm, as the context length $k$ places a strict upper bound on the number of comparisons we must perform when attempting to determine the relative order of a pair of suffixes. Specifically, it follows directly from Theorem 3 that caps-sa performs at most $\mathcal {O}\left( n \log n + nk\right)$ character comparisons, in the worst case, when constructing the context-bounded sa of order $k$.

**Fig. 4:**
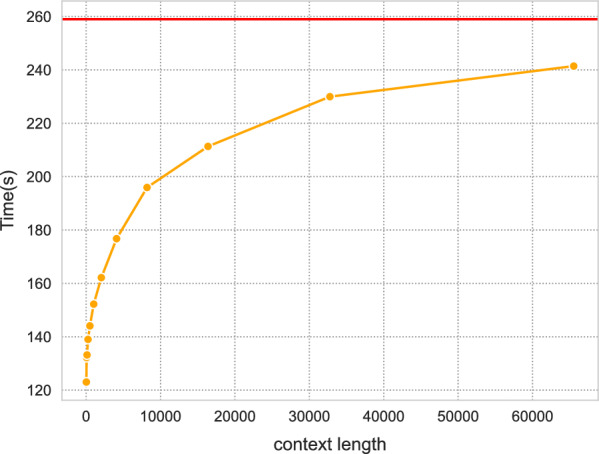
Runtimes of caps-sa as context size increases on the bacteria (1K) dataset. Small context sizes greatly diminish the running time of the algorithm, but runtime is less sensitive for larger context sizes. The full-context runtime is shown as the red horizontal line

In Fig. 4, we report the timing requirements to construct the context-bounded sa of varying orders of powers of $2$, from $64$ to $65,\,536$, over the Bacteria (1K) dataset. As expected, the bounded-context sas can be constructed substantially faster than the full-context sa.

## Conclusion

In this manuscript, we introduced a new method, caps-sa, for parallel sa and lcp-array construction. caps-sa displays very good cache performance (i.e. very low cache miss rate), and scales well to many threads. As a result, caps-sa is able to outperform existing state-of-the-art parallel sa construction algorithms like parallel-divsufsort and parallel-dc3 on genomic datasets. At the same time, caps-sa is substantially simpler than existing state-of-the-art algorithms. This simplicity eases implementation, and leads to many opportunities for further future improvements. Likewise, caps-sa provides the lcp-array directly as a byproduct of sa construction, and does not require a separate algorithm to produce this useful auxiliary data structure. We hope that will prove to be useful in utilities where parallel sa construction is a core subproblem, and also hope that the relatively straightforward algorithm will benefit from further optimizations, enhancements, and alternative implementations within the community.

As caps-sa scales well with the level of available parallelism, and performs well for large references, we expect that it will provide a useful option for tools that seek to build the sa in parallel environments. In addition to the *time* taken to construct the sa or the $LCP$-array, another consideration is the memory (specifically the RAM) required for construction. One approach to improve the *memory-scalability* of sa construction algorithms is to develop external-memory construction algorithms. For example, pSAscan [28] is a state-of-the-art external-memory algorithm for sa construction. Such approaches make use of external-memory (i.e. disk) and algorithms that access and construct the sa in a structured way are likely amenable to external-memory variants.

We note that, though we have not explored it in this manuscript, caps-sa is highly-amenable to external memory implementation. This is because the initial partitioning generates many small subproblems that can be solved independently — i.e. some subproblem can be paged into RAM while others remain on disk. Pivot sampling from the subproblems can be done through a similar paging process. Likewise, after pivot selection, many approximately equal-sized partitions will be created, and these sub-problems, which target specific output intervals of the final suffix array, can be solved independently and in parallel with the relevant data for only a working subset of partitions paged into RAM with the remaining partitions residing on disk. Further, given a sufficiently fine-grained partitioning, the algorithm can likely provide tight controls on the required working memory. As more RAM use is allowed, a larger number of partitions will be allowed in RAM at once, and our algorithm will be able to better make use of available parallelism. On the other hand, as the maximum allowed RAM usage is restricted, fewer partitions will be present in memory at once, potentially limiting parallelism, but adhering to the requested RAM constraints. In practice, we believe that, so long as a sufficiently fine-grained partitioning is used, external-memory variants of our algorithm will still be able to efficiently make use of many threads while still substantially reducing the required working memory. We leave the efficient implementation of an external-memory variant of caps-sa to future work.

## Data Availability

The implementation is available under an open-source license at https://github.com/jamshed/CaPS-SA.

## Appendix

Methods

Pseudo-codes for the procedures absent in the main text, sample-pivots, collate-partitions, and compute-boundary-lcps are provided in the following.

## Results

See Table 4

**Table 4:** Time- and memory-performance results for constructing sas (and lcp-arrays in case of caps-sa) sequentially, i.e. with 1 thread

Dataset	Divsufsort	Parallel-divsufsort	Parallel-dc3	Caps-sa
Human (GRCh38)	9 (25)	29 (32)	36 (110)	35 (45)
Human (T2T)	10 (26)	32 (34)	38 (115)	34 (48)
CdBG (Human reads)	12 (33)	36 (44)	48 (172)	39 (61)
Great white shark	13 (36)	43 (47)	51 (185)	43 (65)
Bacteria (1K)	726 (33)	3300 (43)	3075 (172)	5294 (60)

divsufsort is shown as the optimal serial benchmark. Time is reported in minutes, and the memory usages are reported in GBs in parentheses

## Footnotes

1 With the special case of $L_0 = 0.$

2 $\mathcal {O}(sp \log {sp} + \sum _{i = 1}^{sp} {L_p}_i)$ comparisons are performed, where $L_p$ is the lcp-array of the sorted samples.

3 The *worst-case* work is $\mathcal {O}(n^2)$ due to the second factor. The first factor does not change.

4 https://www.ncbi.nlm.nih.gov/grc.

## Data availability

All the datasets used in this manuscript are publicly available, and have been cited appropriately.
